# Adjustable-rate mortgages in the era of global reflation: How to model additional default risk?

**DOI:** 10.1371/journal.pone.0263599

**Published:** 2022-03-21

**Authors:** Ádám Banai, Edina Berlinger, Barbara Dömötör

**Affiliations:** 1 Executive Directorate for Monetary Policy Instruments and Foreign Reserve Management, National Bank of Hungary, Budapest, Hungary; 2 MNB Institute, John von Neumann University, Kecskemét, Hungary; 3 Department of Finance, Corvinus University of Budapest, Budapest, Hungary; BeiHang University School of Economics and Management, CHINA

## Abstract

We investigate the problem of interest rate risk transforming into default risk of adjustable-rate mortgage loans in the EU. Bank regulation is strikingly not neutral in this aspect, it explicitly favors short-duration adjustable-rate loans over long-duration fixed-rate loans in the framework of the gap management. This asymmetry in the regulation creates perverse incentives both for banks and households, which can lead to aggressive risk-taking, over-indebtedness of unhedged households, high procyclicality of mortgage markets, and increased systemic risks. We present a stress test model to quantify potential losses stemming from this specific risk from the perspective of lender institutions. We estimate the average extra capital that is needed to cover the additional risk of adjustable-rate mortgage loans in the EU to be 0.53% of the value of the total mortgage portfolio and 1.97% of the value of the adjustable-rate mortgage portfolio. We propose introducing a stress test model as a new mandatory element into banks’ risk management framework.

## 1. Introduction

Foreign exchange (FX) lending for households or other unhedged borrowers has been widely discussed in the literature. In this context, Eichengreen et al. [1] called indebtedness in foreign currency the original sin. Several authors concluded that FX debts of unhedged borrowers may threaten financial stability, worsen the effectiveness of the monetary policy, and lead to severe social crises [2–4]. Transferring unmanageable risks to households is contrary to the basic principles of sustainable finance [5].

The risk of adjustable-rate mortgages (ARMs) for the household sector has been subject to much lower attention in the literature although it is similar to FX lending in several aspects. A huge and mainly underestimated market risk (changes in interest rates or FX rates) can easily turn into credit risk and lead to significant credit losses in mortgage markets. There are different channels through which interest rate risk may increase the delinquency rate of mortgages. As Doms et al. [6] showed in their paper for the US market during the Great Financial Crisis (GFC), rising interest rates led to mass defaults of subprime mortgages due to higher installments. Higher installments worsen the ability to pay mortgages but also have a negative effect on willingness-to-pay since higher rates may decrease the potential appreciation of house prices. Pennington‐Cross and Ho [7] also found that default probabilities increase dramatically in the case of low or negative home equity. From this perspective, widespread ARMs—similarly to widespread FX lending—may also narrow down the room for maneuver of monetary policy since any interest rate decision can have serious consequences for financial stability and social cohesion. This is especially true in today’s environment, where after many years of zero rates, central banks started to tighten monetary conditions. Although the ECB and the FED are still waiting, sooner or later they will start raising interest rates. Many central banks of smaller EU countries are already in their hiking cycle.

Our paper fills out a research gap from several aspects. First, we contribute to the literature on the choice between adjustable-rate (ARM) versus fixed-rate (FRM) mortgages by investigating an important effect on the supply side that has not been discussed so far, namely banks’ capital regulation. We show that capital adequacy regulations are distorting towards ARM lending. Second, we propose a specific stress test supplement to the Basel regulations which would make capital calculations neutral from the ARM-FRM perspective. Our recommendation builds on the literature of credit and market risk management, especially the modeling of wrong-way risks. Finally, we elaborate on a stress test methodology by which we quantify the extra default risks and the corresponding extra capital need of ARMs relative to FRMs for the EU banking sector. Our empirical study draws attention to the significant unhedged household exposure in many European countries.

Borrowers find ARM loans more attractive if the yield curve is increasing (so most of the time) as they focus on short term cost minimization while underestimating the future interest rate risk [8–11]. Berlinger [12] argued that the calculation method of APRC misleads the borrowers and strengthens their myopic view. Several authors found that personal traits and psychological effects also play a role in borrowers’ choice [13–15], while Albertazzi et al. [16] and Gathergood and Weber [17] emphasized the effects of education and financial literacy. The product characteristics are more complicated in the case of ARMs as compared to FRMs since installments are not a linear function of interest rates [18]. The National Bank of Hungary [19] found that customers do not understand the risks of variable-rate mortgages as an overwhelming majority of borrowers could not answer what would happen with the installment after a rate hike. Therefore, with ARM loans, a large amount of interest rate risk is transferred from professional banks to non-professional households that can hardly calculate, hedge, or manage it. After a decade of a low interest rate environment, it has become increasingly important to get prepared for the potential broader scale of defaults.

Moench et al. [20] and Zocchi [10] pointed out that the share of ARM loans depends heavily on supply-side factors, as well, most importantly the availability of securitization and state guarantee programs for the banks. Foà et al. [21] documented that banks hit by shocks (related to the deposit base, long term funding, or securitization) tend to lend ARMs; and this can be effectuated through different channels such as the pricing of loan products and distorted advice to borrowers. In connection with this research line, we draw attention to another significant aspect: the risk regulation of banks. Financial regulation prescribes the measurement of the sensitivity of the bank’s economic capital to an interest rate change by calculating the duration of assets and liabilities and the duration gap reflecting the asset-liability mismatch of the institution. As loans provided by a bank usually have a longer tenor than the maturity of the deposits, banks can reduce the duration gap, hence capital requirements by providing ARM loans with shorter interest rate periods, thereby reducing their exposure to market risk.

While the optimal mortgage contract is not a straightforward issue either at an individual or social level [22,23], empirical studies found that ARM loans have significantly higher credit and systemic risk than FRM loans [6,7,24]. Campbell and Cocco [24] modeled mortgage markets including the potential effects of interest rate hikes. According to their results, default rates are the highest for ARMs originated at low interest rates. This mechanism was confirmed by the empirical default rates of the US mortgage market. Moreover, ARM loans defaulted significantly more frequently in the 2000s, too, in a period of decreasing interest rates, which can be explained by the fact that ARMs attract borrowers with the highest income risk. Gaudêncio et al. [25] found the same relation between ARM loans and default probability in EU countries.

In the case of ARM loans, market risk (a rise in interest rates) can easily translate into credit risk (a rise in default rates). Financial regulation, however, does not handle this interconnection properly as according to the classical silo-based approach, different risk types are measured and managed separately. Risk assessment improved after the GFC in many aspects, and the regulation called for banks to assess all types of unexpected and expected losses insufficiently covered by provisions [26]. It also addresses the interconnection of credit and market risks by introducing the concept of wrong-way risk deriving from the positive correlation between the probability of default and market variables (defined as general wrong-way risk); however, in practice, it is applied only to derivative transactions. The regulation also highlights the importance of stress testing that aims to identify potential losses and liquidity needs under adverse circumstances [27], but no mandatory stress scenarios are prescribed to capture interactions between market and credit risks.

In this paper, we propose a stress test model to measure the extra risk arising from ARM lending in the EU banking sector. We estimate the default risk hidden in ARM loans quantifying the potential losses on ARMs stemming from a hypothetical rate hike (2%). We find that at the EU level, on average, the potential loss due to this specific risk can be 0.53% of the total value of the mortgage portfolio, which is 1.97% for the ARM loan portfolio under a realistic scenario; but results are highly sensitive to the assumptions and parameter settings.

The paper follows the following structure: The next session describes the regulation of market and credit risks; then, we present the stress test model calibrated to the banking sectors of EU countries; finally, we summarize our main findings and conclusions.

## 2. Risk management of ARM loans

Originally, financial regulation required banks to quantify their credit risk, market risk, and operational risk separately. The minimum capital requirement was calculated by simply adding up the capital requirements of the different risk types. The need for integrated measurement of market and credit risk appeared around 2010. Bunn et al. [28] showed that since income is an important determinant of both corporate and household probability of default (PD), macroeconomic variables affecting income gearing (payment-to-income ratio) have credit loss consequences as well. Alessandri and Drehmann [29] and Drehmann et al. [30] modeled the risk of a hypothetical banking book under different stress scenarios incorporating the dependency of credit risk on interest rate shocks. They concluded that the interaction is significant between interest rate risk and credit risk, so neglecting this effect would lead to an underestimation of risks. Kupiec [31] derived a model in which a common Gaussian factor impacts migration-style credit risk as well as market risk. In this model, the portfolio’s future value was calculated using a Monte Carlo simulation and he found that due to the complex effects, an integrated model can give higher or lower results than the sum of the parts. Kretzschmar et al. [32] suggested an integrated economic scenario-based model where the price changes of the bank’s assets were driven by common macroeconomic factors. They showed that implementing an integrated risk model can help to avoid the undercapitalization of banks. Therefore, if the banking portfolio is simultaneously affected by market risk and credit risk factors, an integrated risk assessment is needed for the prudent operation. Breuer et al. [33] also showed an example of foreign currency loans where the integrated risk capital was significantly larger than the sum of the capital calculated for market and credit risks separately at all significance levels.

A new element in the Basel regulation is the concept of wrong-way risk which captures the risk deriving from the fact that the parameters of the loss distribution are not independent of each other. BCBS [27] distinguishes two subcategories: general and specific wrong-way risks. A general wrong-way risk arises when the probability of default of counterparties is positively correlated with general market risk factors; while specific wrong-way risk refers to the situation when the exposure to a particular counterparty positively correlates with the probability of default of the counterparty due to the nature of the transactions with the partner. The regulation requires banks to increase the credit value adjustment for financial derivatives due to the specific wrong-way risk and to conduct stress tests to measure the effect of the changes in the risk factors or in their correlations. However, there is no explicit method or scenario to be used for the wrong-way risk reporting for credit portfolios.

In practice, market risk can translate into credit risk also in the case of mortgage portfolios. A change in the interest rate, which is a kind of market risk, influences the economic value by repricing the bank’s assets and liabilities. The change of the economic value depends on the extent of the actual yield change and the duration gap of the institution. Simultaneously, the payments of the ARMs will also be recalculated, so the payment-to-income ratio of the borrowers will change. The probability of default has a strong connection to mortgage income gearing. In the Bank of England’s model, a 1% change in the mortgage income gearing caused a 0.3 percentage point change in the arrears rate [28]. Balás et al. [34] analyzed the factors affecting the probability of default (PD) empirically and they found a strong relationship, too. A change of 1 percentage point in the payment-to-income ratio caused a 0.76 percentage point increase in the default rate. A sudden jump in the PD may lead to a hike in the rate of non-performing loans; however, it also implies an increase in the capital requirement of the whole loan portfolio as a higher PD causes an increase in the worst-case default rate.

The Basel regulation does not explicitly address the additional credit risk of ARM loans, hence, depending on the local banking and regulatory practices, this specific risk might be entirely or partly neglected. Banks’ ARM risks can be managed by prescribing stricter eligibility criteria for ARM loans (e.g., lower payment-to-income (PTI) or loan-to-value (LTV) ratios at issuance), establishing extra provisions, and providing extra capital in the framework of the internal capital adequacy assessment process (ICAAP). Corresponding to the last point, we propose a stress test methodology designed specifically to capture the interaction between the interest rate risk and default risk of ARM loans.

In the following section, we present a stress model to quantify the potential extra capital need of the European banks at a country level. A similar method could be used at a bank level as well. Applying a stress test like this and calculating the capital requirement accordingly may counterbalance the incentives to hedge the duration gap by offering ARM loans.

## 3. Stress model

To estimate the additional credit risk of ARM loans (relative to FRM loans) in the European banking sector, we use data of the European Mortgage Federation (EMF) published in Hypostat [35] on the developments in the housing and mortgage markets in Europe and beyond with a special focus on the differences in interest rates, maturities, portfolio values, and shares of ARM loans.

According to the regulation on prudent banking, all relevant risks should be modeled, measured, mitigated, and monitored. For the expected losses, banks establish provisions and make price adjustments, while for the unexpected losses (difference between the expected loss and some downside risk threshold), they provide capital [27].

In the case of ARM loans, too, the additional default risk can be decomposed into expected and unexpected parts. Using the expected change in interest rates, we can estimate the expected loss that should be reflected in the provisions and in the pricing of ARM products. However, in the low-rate environment we live in, the expected rise in interest rates (calculated on a historical basis) is close to zero even for a longer time horizon, so the expected ARM-specific default loss can be considered zero. In contrast, in practice as inflation expectations are increasing in all countries and some central banks already made their first tightening steps, we must prepare for large interest rate hikes too.

In this section, we develop a specific stress test model to quantify the additional default risk and the corresponding additional capital need if a bank lends ARM (instead of FRM), ceteris paribus.

A stress test is a semi-quantitative method of risk analysis, which means analyzing the effects of possible outcomes without assigning probabilities to these outcomes. Fully quantitative methods, on the other hand, rely on a complete probability distribution, so all possible outcomes and their associated probabilities should be determined first, and then some risk measures (Value-at-Risk, VaR; Expected Shortfall, ES; etc.) can be calculated [36,37].

Stress test methodology fits better our research than fully quantitative methods for several reasons. First, in this way, we do not have to forecast future changes in the interest rates, hence, we don’t have to model their stochastic processes. This is an advantage because currently, there is a great deal of uncertainty in forecasting interest rates; past interest rates cannot be projected into the future due to, for example, large regime changes (impacts of the Covid crisis, crisis management, inflation, etc.). In our stress test, we assume a severe but still possible change in the interest rates and quantify its effect, but we do not need to specify the probability of this event. The only requirement is that the assumed stress scenario (the change in the interest rate) should be realistic. Second, according to the GFC-induced new regulatory recommendations, more emphasis should be put on stress testing rather than on the mechanistic application of quantitative (VaR, ES) models [38,39]. These latter quantitative models rely inevitably on historical data, hence, are backward-looking. In contrast, stress testing, if properly done, can be more forward looking, and risk management should be concerned with the future. Financial regulators highly recommend stress testing also because it supports internal and external communication and facilitates risk mitigation or contingency planning [39]. Third, the stress test methodology suits well the framework of the Internal Capital Adequacy Assessment Process (ICAAP) in the second pillar of banking risk management. Stress test tools have been specifically developed to look at how negative scenarios affect the underlying factors that ensure safe operation and to assess the shock-resilience of institutions, hence their contribution to the systemic risk [40]. For banks, the classic regulatory and supervisory requirement is to have the right amount of capital to ensure the repayment of liabilities even in a stress event. Standardized liquidity and capital rules are also rooted in a stress test logic. Moreover, stress tests are widespread in other industries as well when analyzing fundamental risks.

The logic of the proposed stress test model is the following:

$$\Delta r\to \frac{\Delta C}{C}\to \Delta PD\to \Delta CR$$ (1)

where Δ*r* is the assumed change in the interest rates in an extreme but plausible stress scenario over a holding period of 1 year; $\frac{\Delta C}{C}$ is the percentage change in the mortgage repayment; Δ*PD* is the increase in the probability of default relative to the base case (with no change in the interest rate level), and *ΔCR* is the additional capital need of ARM loans relative to FRM loans.

The definition of a stress event for a single market variable or for the whole market is not straightforward [41]. Here, in line with the Basel stress methodology [27], the assumed extreme change in the interest rate Δ*r* is 2%. This is consistent also with the EBA guideline where the macro-financial stress scenario is considered with an increase of 2.5% in the interest rates over 3 years [42].

According to Berlinger [12], the repayment of ARM loans changes in line with the change in the interest rate, and the interest rate sensitivity equals the modified duration D* of a similar but FRM loan:

$$\frac{\Delta C}{C}=D^{*}\Delta r$$ (2)

We aggregate all residential loans for a country and characterize them with the typical interest rate *r*, typical maturity at issuance *M*, the total amount of outstanding mortgage loans *V*, and share of ARM loans *a*. We assume, for the sake of simplicity, that for a given country, parameters *r*, *M*, and *a* are the same for each year cohort *y* (loans issued in year *y*); and due to the lack of year-specific data, the nominal values of new loans are also supposed to be the same over time. So, we assume stationary year cohorts in all relevant aspects. Therefore, the interest rate sensitivity of the total outstanding mortgage portfolio D* in (2) is given by

$$D^{*}=\frac{1}{1+r}\sum_{y=0}^{M-1}D_{y}w_{y}$$ (3)

where *D*_*y*_ and *w*_*y*_ are the duration and the relative weight of the *y*th year cohort’s portfolio, respectively. We calculate the duration of a similar but fixed mortgage portfolio for each year cohort *D*_*y*_ as

$$D_{y}=\frac{1+r}{r}-\frac{T}{{\left(1+r\right)}^{T}-1}$$ (4)

where *T* is the remaining maturity.


T=M−y (5)

Relative weights are defined as

$$w_{y}=\frac{V_{y}}{\sum_{y=0}^{T}V_{y}}=\frac{V_{y}}{V}$$ (6)

where, *V* is the present value of the outstanding loan portfolio, and indices refer to the year of issuance. We model only one repayment per year, which simplifies calculations without the loss of generality:

$$V_{y}=C_{y}AF(r,T_{y})$$ (7)

where *AF*(*r*, *T*_*y*_) is the annuity factor depending on interest rate *r* and remaining maturity *T*_*y*_. As year cohorts are stationary, initial loan amount *L*, initial maturity *M*, and, hence, initial repayment *C* remain the same in each cohort:

$$C_{y}=C=\frac{L}{AF(r,M)}$$ (8)

Once we calculated the increase in the repayments $\frac{\Delta C}{C}$ using (2), we estimate the corresponding increase in the probability of default *PD*. We assume a sigmoid *PD* function, which is consistent with the probit model widely used for modeling default losses, the KMV method [43], and the results of the agent-based simulation model of Campbell and Cocco [24] as well:

$$PD=N\left(\frac{\Delta C}{C}\right)=N(\mu ,\sigma )$$ (9)

**Fig 1:**
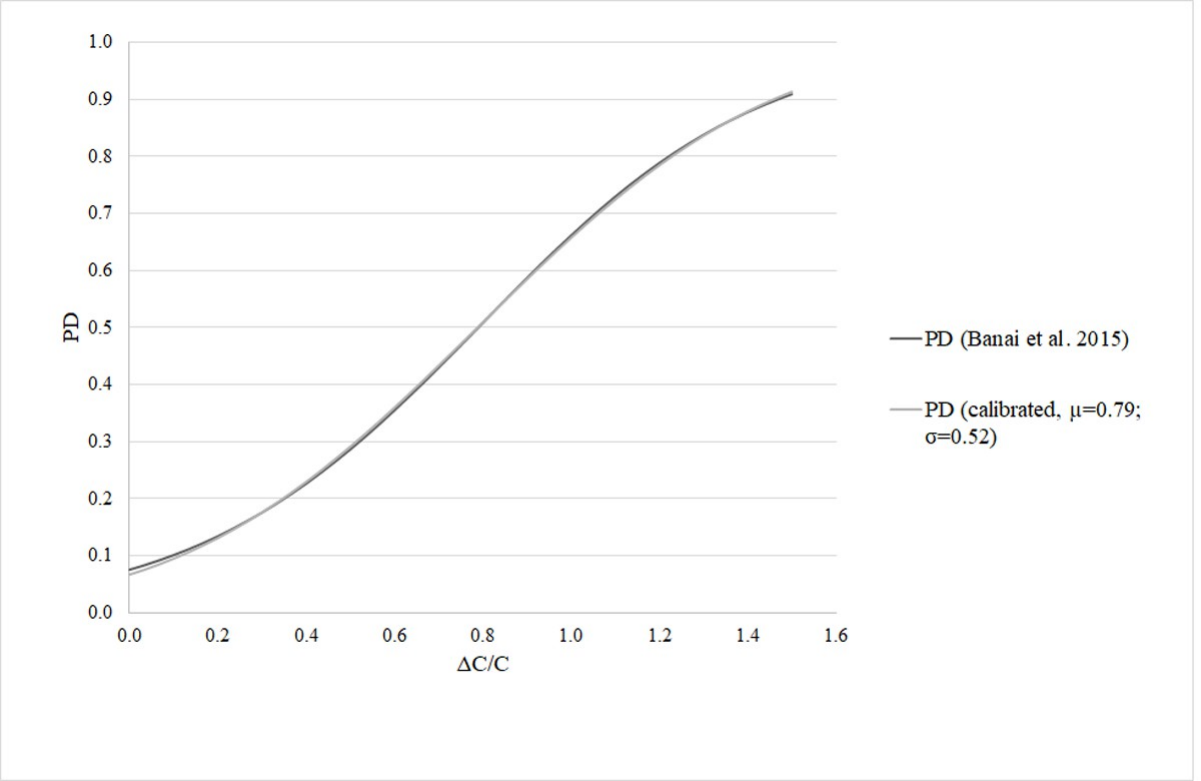
Probability of default in the function of the percentage change in mortgage payments. Fig 1 presents the empirical *PD* function in function of the percentage change in the repayment *C* based on [34] and the calibrated *PD* curve we use in the stress test. The two curves are very close to each other.

In (9), *N* stands for a cumulative normal distribution function with a mean *μ* and a standard deviation *σ*. Parameters *μ* and *σ* are latent variables depending on the characteristics of the ARM portfolio. The mean *μ* reflects the average riskiness of the borrowers (how much they are indebted relative to their income on average), and *σ* reflects the heterogeneity of borrowers in terms of their riskiness. We calibrate our *PD* function (9) to the *PD* function estimated by Balás et al. [34, Chart 2] for borrowers with average income indebted in home currency in the Hungarian mortgage market assuming an initial payment-to-income ratio of 30%. Given that Balás et al. [34] analyzed real-life microdata, their *PD* function can be considered as a realistic estimation. Minimizing the sum of squares of the differences, we get *μ* = 0.79 and *σ* = 0.52 (Fig 1).

Using a sigmoid *PD* function, we implicitly assume that at an individual level, there is a threshold in the loan repayments which triggers default. So, also at a portfolio level, there can be a sharp transition from a regime of few defaults to many defaults as required loan repayments increase, hence *PD* is not linear in $\frac{\Delta C}{C}$ either at an individual or an aggregate level. Clearly, *PD* is highly dependent on parameters *μ* and *σ*. In our analysis, first, we take the calibrated parameters (*μ* = 0.79 and *σ* = 0.52) as a basis and estimate default losses accordingly. Later, we run a sensitivity analysis for these model parameters.

When calculating the additional capital need for ARM loans *ΔCR*, we use the following formula:

ΔCR=EAD∙LGD∙ΔPD (10)

where *EAD* is the exposure at default which equals the present value of the mortgage portfolio affected by the increase of the interest rate, and *LGD* is the loss given default (in percentage), Δ*PD* stands for the change in the PD relative to the base case ($\frac{\Delta C}{C}=0$) Note that *ΔCR* is the additional capital need designated to cover the ARM-specific additional credit risk due to the potential increase of interest rates.

Country-level mortgage portfolios in the EU are built up from loans of different interest rate adjustment periods. Loans with an extremely short interest rate period (up to 1 year) are considered as ARMs, while for the rest, interest rates are supposed to be fixed for the whole maturity (FRM). Thus, our estimation for the additional capital need can be considered as a lower bound. To quantify the effect of increased *PD*s, we consider only ARM loans (up to 1 year); therefore, *EAD* is calculated as

EAD=V∙a (11)

Using (2), (9), (10), and (11), the additional capital need *ΔCR* for a country is

$$\Delta CR=EAD∙LGD∙\Delta PD=V∙a∙LGD∙(N\left(\frac{\Delta C}{C}\right)-N(0))=V∙a∙LGD∙(N(D^{*}\Delta r)-N(0))$$ (12)

To have comparable values across different countries, we divide Δ*CR* by the total value of the mortgage portfolio *V* or the value of the ARM portfolio *V*∙*a*.

Having no access to country-specific loss given default ratios *LGD* for mortgage portfolios, we set it uniformly at 35%—the lowest possible value suggested by the EU’s Capital Requirement Regulation (CRR) [44] for exposures with eligible collaterals.

Relying on our stress methodology (12) and the database of Hypostat [35], we estimate the ratios of the additional capital need for each member of the EU (the UK included) in November 2020. Table 1 presents the input data.

**Table 1:** Characteristics of mortgage markets in EU countries UK included), November 2020.

Country	Typical mortgage rate	Typical maturity	Total outstanding residential loans	Share of ARM loans
	*r*	*M*	*V*	*a*
	(%)	(year)	(M euro)	(%)
Austria	1.6	27	119,775	43.67
Belgium	1.8	20	263,419	6.36
Bulgaria	3.3	20	6,384	99.01
Croatia	3	25	7,720	13.32
Cyprus	2.1	22	8,605	93.22
Czechia	2.6	25	48,658	18.40
Denmark	0.7	30	258,799	33.40
Estonia	2.5	30	8,119	89.88
Finland	0.7	25	100,354	93.30
France	1.3	20	1,078,000	0.00
Germany	1.5	25	1,530,434	11.00
Greece	3.1	25	52,707	83.92
Hungary	4.9	15	13,715	49.00
Ireland	2.9	25	81,637	78.60
Italy	1.4	22	382,583	29.36
Latvia	2.7	20	4,177	95.78
Lithuania	2.4	30	8,411	98.39
Luxembourg	1.4	27	35,633	38.95
Malta	2.6	25	6,071	44.41
Netherland	2.3	30	722,672	18.72
Poland	4.3	30	108,382	100.00
Portugal	1.2	33	93,846	71.75
Romania	2.5	30	16,999	77.00
Slovakia	1.4	25	31,001	1.71
Slovenia	2.4	20	6,587	52.76
Spain	2	25	487,561	35.64
Sweden	1.5	40	422,742	67.00
UK	1.8	25	1,708,134	31.60

Source: Hypostat [35].

We can see in Table 1 that EU countries are highly different in terms of mortgage rate *r*, maturity *M*, the total value of mortgage portfolios *V*, and share of ARM loans *a*.

Table 2 presents the output of our stress test, assuming an upward parallel shift in mortgage rates of Δ*r* = 2% and using the calibrated *PD* function.

**Table 2:** Results of stress testing for EU countries (UK included), November 2020.

Country	Interest rate sensitivity of ARM loans	Percentage change in payment	Exposure at default (M euro)	Change in the probability of default	Additional capital requirement (M euro)	Relative additional capital requirement (to the total portfolio)	Relative additional capital requirement (to the ARM portfolio)
	D*	*ΔC/C*	*EAD*	ΔPD	*ΔCR*	*ΔCR/V*	*ΔCR/Va*
Austria	8.76	0.18	52,306	5.5%	999	0.8%	1.9%
Belgium	6.75	0.13	16,753	4.0%	234	0.1%	1.4%
Bulgaria	6.42	0.13	6,321	3.8%	83	1.3%	1.3%
Croatia	7.76	0.16	1,028	4.7%	17	0.2%	1.6%
Cyprus	7.23	0.14	8,022	4.3%	121	1.4%	1.5%
Czechia	7.89	0.16	8,953	4.8%	150	0.3%	1.7%
Denmark	9.99	0.20	86,439	6.4%	1,943	0.8%	2.2%
Estonia	9.18	0.18	7,297	5.8%	148	1.8%	2.0%
Finland	8.51	0.17	93,630	5.3%	1,726	1.7%	1.8%
France	6.86	0.14	0	4.1%	0	0.0%	0.0%
Germany	8.24	0.16	168,348	5.1%	2,984	0.2%	1.8%
Greece	7.73	0.15	44,229	4.7%	725	1.4%	1.6%
Hungary	4.88	0.10	6,720	2.7%	64	0.5%	1.0%
Ireland	7.79	0.16	64,167	4.7%	1,062	1.3%	1.7%
Italy	7.42	0.15	112,326	4.5%	1,754	0.5%	1.6%
Latvia	6.55	0.13	4,001	3.8%	54	1.3%	1.3%
Lithuania	9.23	0.18	8,276	5.8%	169	2.0%	2.0%
Luxembourg	8.84	0.18	13,879	5.5%	268	0.8%	1.9%
Malta	7.89	0.16	2,696	4.8%	45	0.7%	1.7%
Netherland	9.27	0.19	135,284	5.9%	2,771	0.4%	2.0%
Poland	8.44	0.17	108,382	5.2%	1,977	1.8%	1.8%
Portugal	10.59	0.21	67,335	6.9%	1,630	1.7%	2.4%
Romania	9.18	0.18	13,089	5.8%	265	1.6%	2.0%
Slovakia	8.27	0.17	530	5.1%	9	0.0%	1.8%
Slovenia	6.61	0.13	3,475	3.9%	47	0.7%	1.4%
Spain	8.08	0.16	173,767	4.9%	3,006	0.6%	1.7%
Sweden	12.26	0.25	283,237	8.4%	8,281	2.0%	2.9%
UK	8.15	0.16	539,770	5.0%	9,443	0.6%	1.7%

Remark: Here, Δr = 2%, μ = 0.79, σ = 0.52, and LGD = 0.35.

**Fig 2:**
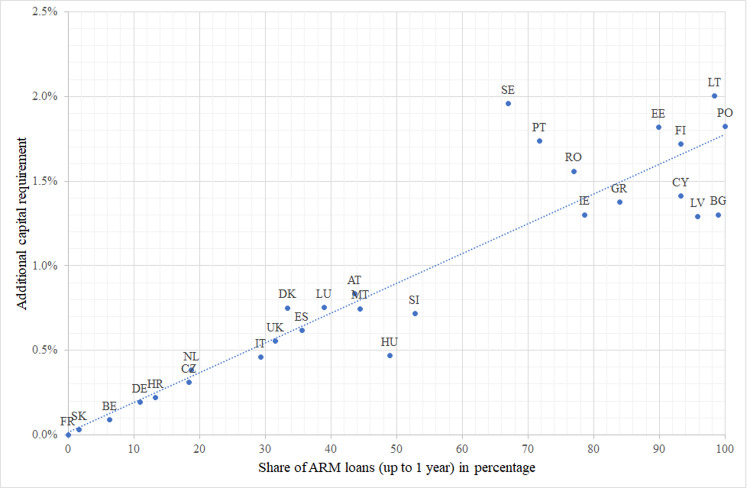
Additional capital need relative to the total portfolio value ΔCR/V in EU countries, November 2020. The additional capital need depends mostly on the share of ARM loans. Due to other parameters (interest rates, maturities, etc.), some countries might be above or below the trendline.

The additional capital need relative to the total value of the mortgage portfolio *ΔCR/V* ranges from 0–0.1% (Belgium, France, and Slovakia) to 2.0% (Lithuania, Sweden); the weighted average is 0.53%. Differences are due to many factors, most importantly, the differences in the shares of adjustable-rate loans, see Fig 2.

**Fig 3:**
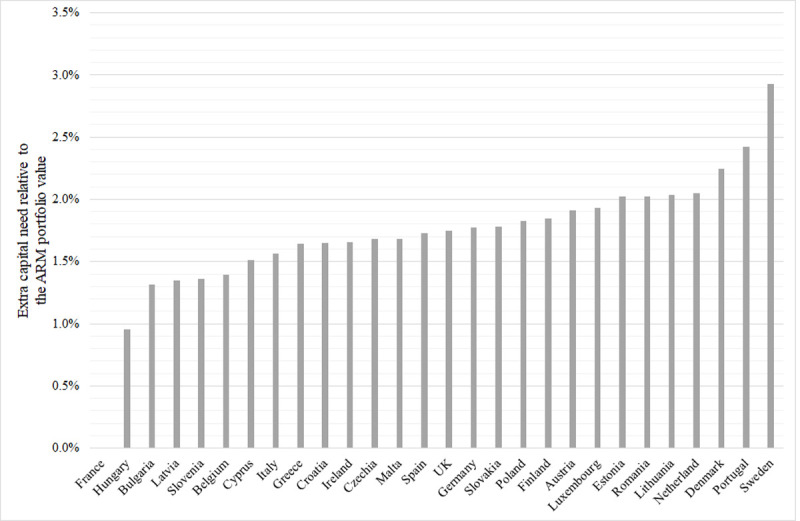
Additional capital need relative to the ARM portfolio value ΔCR/Va in EU countries November 2020. Except for France (where ARM loans with extremely short interest periods do not exist), the additional capital need relative to the value of the ARM portfolio is between 1.0% (Hungary) and 2.9% (Sweden); the average is 1.97%. Differences can be explained mainly by the maturity, which is typically 15 years for Hungary and 40 years for Sweden.

To highlight the effects of other differentiating factors such as maturity and interest rate, we can relate the additional capital need to the value of the ARM portfolio *ΔCR/Va*. Fig 3 presents a bar chart with EU countries arranged according to this ratio.

**Fig 4:**
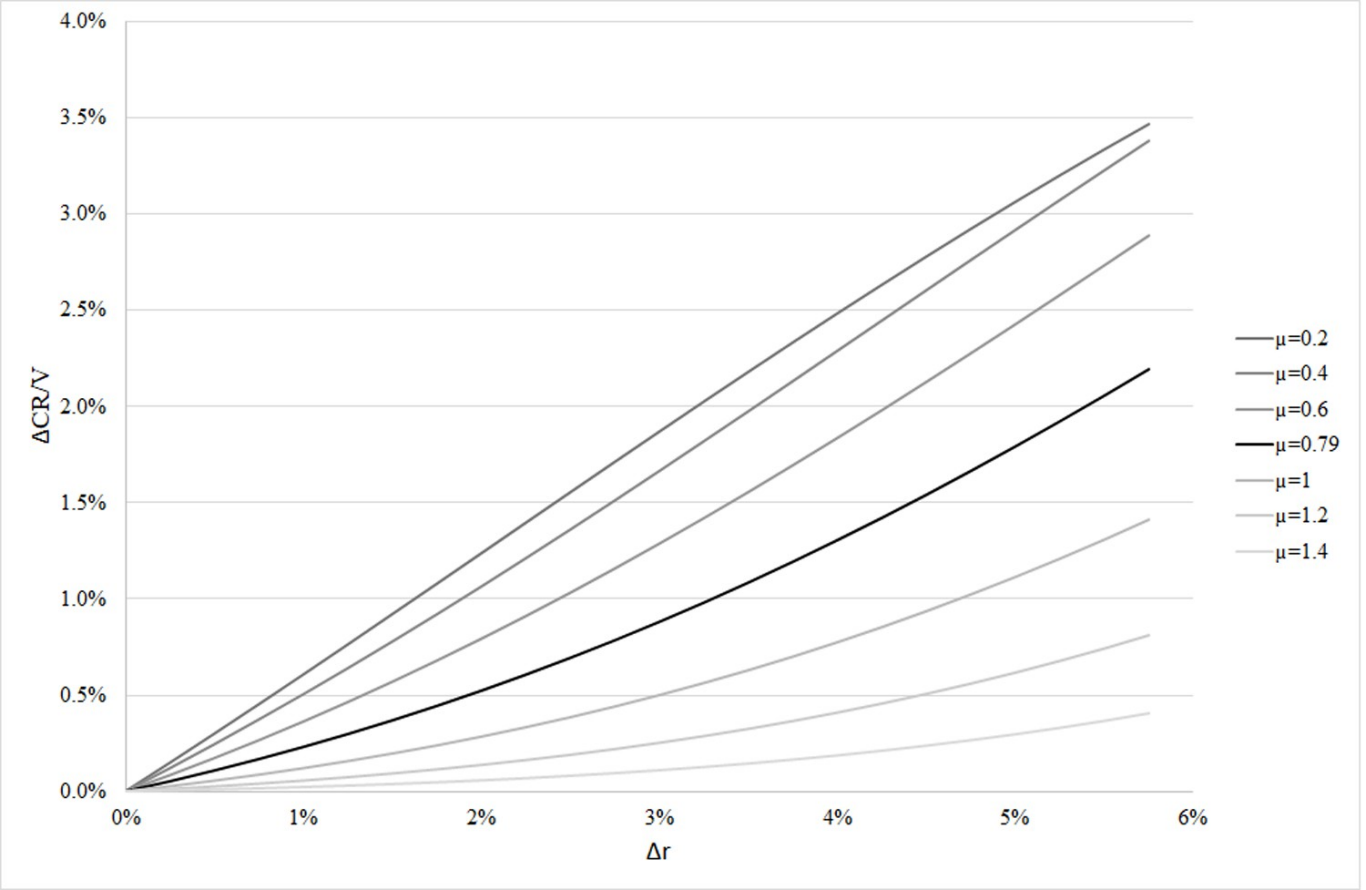
Additional capital need of ARM loans at EU Level as a function of Δr and µ.

**Fig 5:**
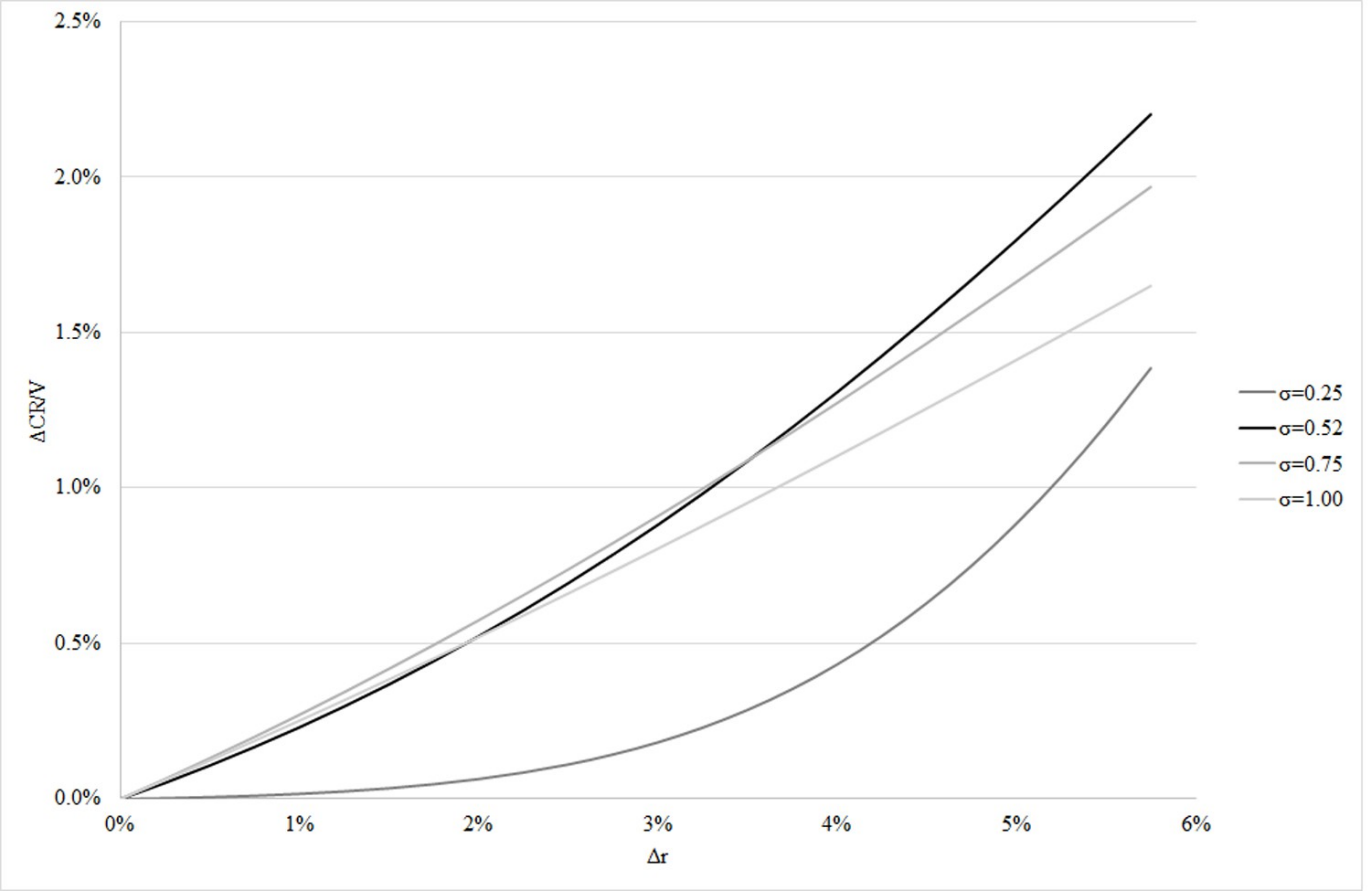
Additional capital need of ARM loans at EU level as a function of Δr and σ. Figs 4 and 5 demonstrate that the calculation of the additional capital need is highly sensitive to the assumed change in the interest (Δ*r*), parameters related to the average riskiness of the mortgage portfolio (*µ*), and inequality of borrowers in their riskiness (*σ*). If loss given default *LGD* differs from our assumptions (0.35), the ARM-specific additional capital will change proportionally.

Clearly, these results are strongly dependent on the assumptions, especially on the potential change in the mortgage rate Δ*r* and the parameters of the *PD* function (*μ* and *σ*). With Δ*r* = 2%, *μ* = 0.79, and *σ* = 0.52, the average additional capital need at an EU level is 0.53% of the value of the total mortgage portfolio (*ΔCR/V* = 0.53%) and 1.97% of the value of the total ARM portfolio (*ΔCR/Va* = 1.97%). Figs 4 and 5 present how the first ratio *ΔCR/V* changes with parameters Δ*r*, *μ*, and *σ*.

Our calculations have some limitations. Most of all, we do not have granulated data on the composition of the loan portfolios related to maturities, interest rates, loan amounts, etc.; therefore, we use averages and make simplifying assumptions (like the homogeneity of year cohorts). Furthermore, we do not know the exact shape of the PD and LGD functions that can vary across banks, countries, and times; thus, our estimations are highly uncertain. Note, however, that we significantly underestimate the ARM-specific extra default risk and the corresponding capital need in the EU banking sector as we overlook ARM loans with interest rate periods of longer than 1 year.

## 4. Conclusions

We focus on the interest rate risk transforming into default risk in the case of ARM loans in the EU banking sector. Although we can witness some convergence in the last decade, there is still surprisingly large heterogeneity in mortgage conditions across the EU [35], which can be attributed to different economic and social conditions, institutions, histories, cultures, etc. In some countries such as Belgium, France, Germany, and Slovakia, ARM loans with an extreme short interest period (up to 1 year) are not widespread at all; hence, the ARM-specific risk is marginal. At the other end of the scale, however, we find countries where aggressive ARM lending is the business standard. The share of ARM loans is the highest in Bulgaria (99%), Cyprus (93%), Finland (93%), Latvia (96%), Lithuania (98%), and Poland (100%).

Bank regulation is strikingly not neutral in this aspect, as it explicitly favors short-duration adjustable-rate loans over long-duration fixed-rate loans in the framework of gap management. Market risks and credit risks are handled separately, and there are no direct prescriptions to quantify interactions between the two. This asymmetry in the regulation creates perverse incentives both for banks and households, which can lead to aggressive risk-taking, over-indebtedness of unhedged households, high procyclicality of mortgage markets, and increased systemic risks.

As capital regulation has become highly complex and contains different stress testing requirements, it may change from bank to bank how they are prepared for losses stemming from the additional credit risk of ARM loans. In any case, as we learned from FX lending, it is difficult to manage the default risk of unhedged borrowers who are not even aware of the risks they are bearing.

We propose a stress test model to estimate potential losses stemming from the specific ARM risk from the lender institution’s perspective. We would like to highlight that although adjustable-rate products seem cheaper and, thus, more attractive for both borrowers and lenders, they can result in higher potential losses. We estimate the average additional capital that is needed to cover the extra ARM credit risk in the EU to be 0.53% of the value of the total mortgage portfolio (the maximal value is 2.0% in Lithuania) and 1.97% of the value of the ARM portfolio (the maximal value is 2.92% in Sweden).

One may think that banks effectively take account of the relationship between macro factors (like inflation) and mortgage defaults when estimating worst-case defaults using advanced default risk models under the first pillar of the Basel regulation. However, as long as ARM and FRM loans are not differentiated in these models, ARM-specific extra risk will not get measured either explicitly or implicitly.

Some banks may model ARM-specific risks under the second pillar of the Basel regulation, the Internal Capital Adequacy Assessment Process (ICAAP), and they may apply an extra multiplier when calculating the regulatory capital if they are heavily involved in ARM lending. However, according to the present regulation, there are no guarantees for this.

In some countries, ARM lending is marginal due to historical, cultural, or institutional reasons; for example, in countries with fewer ARM loans, mortgage portfolios might be safer, so our calibrated PD function may overestimate the corresponding default risk. When developing a tailor-made stress test, parameters can be adjusted to the local conditions taking advantage of the more granulated data.

To some extent, ARM loans may provide an inherent natural hedge against inflation risk, as when inflation rises, interest rates, wages, and collateral values are expected to rise in the longer run. However, correlations are not perfect and there can be significant time lags (which may take years), so, this hedge is far from being perfect [24,25]. Due to the time lag, increasing installments after the rate hike and the inflation of living costs may shock households at the same time. Thus, ARM-specific risks must be properly modeled and measured to ensure a prudent operation.

We can see from the sensitivity analysis that the outputs of the model are highly sensitive to the parameter setting; therefore, the model risk is high. At a portfolio level, however, model risk can be marginal if banks carefully design their own stress methodology and exploit the granulated information they possess in relation to their mortgage portfolio. Our analysis suggests that the upside risk of the capital need can be significant in some countries, so it is advisable to change the structure of the banking book toward fixed-rate contracts.

In many cases, banks have no direct interest in changing their business model and moving toward fixed-rate products. Therefore, we recommend additional regulatory measures, for example, under the second pillar of the Basel regulation, in the ICAAP framework, ARM-specific stress tests focusing precisely on the additional ARM risk should be prescribed. This new supplement would perfectly fit the framework of stress testing already present in the regulation and would significantly contribute to the relevance, completeness, unbiasedness, and consistency of risk management systems.

The macroprudential policy also gives different opportunities to incentivize banks toward fixed-rate lending. The Hungarian National Bank, for example, effectively directed mortgage lending from ARM to FRM by setting stricter eligibility rules for ARM loans (lower PTI and LTV ratios are allowed) and by providing interest rate swaps to make FRM lending more attractive for the banks. As a result, almost 100 percent of newly disbursed loans are fixed [45]. A Systemic Risk Buffer (SRB) may also be a good tool to reflect more on the different risks of mortgages with different fixation. SRB might be applied for ARMs especially if the probability of increasing rates is high.

An important further research topic can be the effect of adjustable-rate loans on SMEs and the lender banks. Adjustable rates may have a similar effect on SMEs as they have on households since they don’t have the deep knowledge to understand the risks and there are no relevant instruments to hedge. It is especially true for smaller, young firms which would like to fund their long-term investments. This consideration at SMEs is reflected by the popularity of the fixed-rate Funding for Growth Scheme in Hungary [45]. The literature also shows the potential negative effect of indebtedness on growth [46], so not only the choice between adjustable and fixed rate is important but the choice between loan and equity.
